# Identification and characterization of G-quadruplex formation within the EP0 promoter of pseudorabies virus

**DOI:** 10.1038/s41598-018-32222-7

**Published:** 2018-09-19

**Authors:** Jiang-Nan kong, Chao Zhang, Yan-Ce Zhu, Kai Zhong, Jiang Wang, Bei-Bei Chu, Guo-Yu Yang

**Affiliations:** College of Animal Science and Veterinary Medicine, Henan Agricultural University, Zhengzhou, 450002 Henan Province PR China

## Abstract

EP0 is an important early gene that modulates the life cycle of pseudorabies virus (PRV). A guanine-rich sequence overlapping with three Sp1 binding sites is located upstream of the transcription start site (TSS) in the EP0 promoter. Using native polyacrylamide gel electrophoresis (PAGE) and circular dichroism (CD), we verified that the G-rich region in the EP0 promoter forms an intramolecular parallel G-quadruplex (G4) in the presence of K^+^ ions. Further dimethyl sulphate (DMS) footprinting and *Taq* polymerase stop assays indicates the potential polymorphic folding of G4. In addition, a small chemical ligand, pyridostatin (PDS), promotes and stabilizes the formation of G4. Interestingly, based on the results of electrophoretic mobility shift assays (EMSA), the Sp1 protein bound to G4-bearing DNA with more affinity than DNA lacking the G4 structure. According to the luciferase reporter assay, G4 negatively regulates the EP0 promoter activity. These results demonstrate that Sp1 and G4 cooperate to regulate EP0 promoter activity.

## Introduction

**Figure 1:**
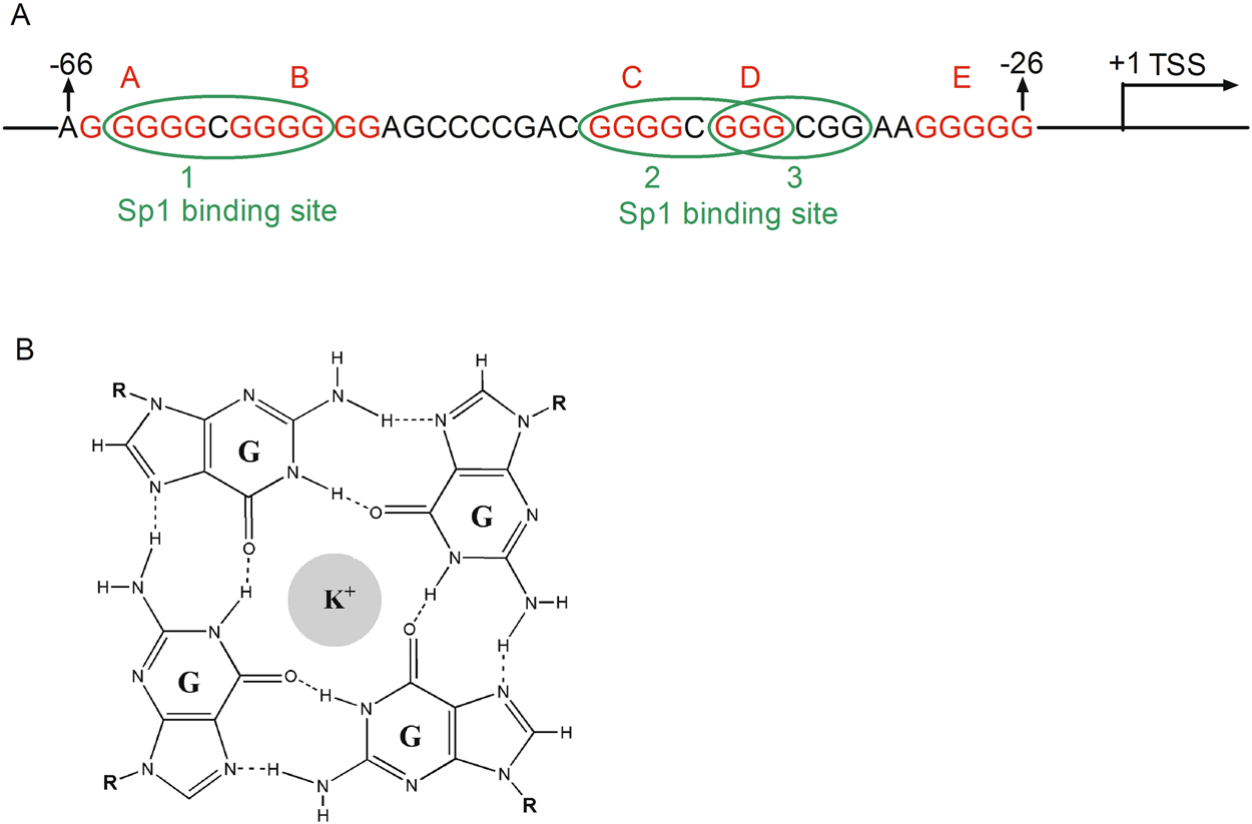
(**A**) Schematic of the conserved G-rich sequence in the EP0 promoter: the G-tracts are shown in red and green ellipses mark the Sp1 binding sites. (**B**) Illustration of a G-quartet formed by four guanines via Hoogsteen hydrogen bonding.

Guanine (G)-rich sequences in DNA or RNA fold into a non-canonical secondary structure named G-quadruplexes (G4)^1–6^, which comprise two or more G-quartets. A G-quartet is a planar array composed of four guanines linked by Hoogsteen hydrogen bonds. The formation of G4 with three tetrads has been extensively studied. However, some papers have reported that two tetrads have capacity to form G4. For example, an extensively studied thrombin-binding aptamer (TBA), 5′-GGTTGGTGTGGTTGG-3′, folds into a quadruplex with two tetrads connected by two TT loops and one TGT loop^4^. In addition, the c-kit^5^ and human thymidine kinase 1 (TK1)^6^ genes containing two tetrads also have been reported to form quadruplexes. G4s are preferentially stabilized by monovalent cations, such as Na^+^ or K^+^ (Fig. 1B). Based on accumulating evidence, putative quadruplex-forming motifs are not only locate at telomeres^7^, immunoglobulin switch regions^8^ and insulin regulatory regions^9^ but are also enriched in the promoters of oncogenes, such as c-MYC^10^, c-KIT^11^, KRAS^12^, BCL2^13^, hTERT^14^, VEGFA^15^, in mammalian cells. The formation of G4s in these promoters typically acts as an on/off switch to regulate gene transcription. In addition, G4 has been detected *in vivo* using an engineered antibody^16,17^. In addition to mammalian cells, G4-forming sequences are present in the genomes of many viruses, such as human immunodeficiency virus-1 (HIV-1)^18^, Epstein-Barr virus (EBV)^19^, herpes simplex virus (HSV)^20^, hepatitis C virus (HCV)^21^, Ebola virus disease (EVD)^22^, Zika virus^23^ and hepatitis B virus (HBV)^24^. Sara N. Richter’s group and other groups have reported that a G-rich sequence in the Nef coding region^25^ and in the unique long terminal repeat (LTR) promoter^18,26,27^ of HIV-1 forms G4. Furthermore, the G4s, aided by some chemical ligands, exert an antiviral effect by inhibiting LTR promoter activity^26,28,29^.

Pseudorabies virus (PRV) is a member of *Alphaherpesvirinae* subfamily and is the causative agent of Aujeszky’s disease or pseudorabies (PR). This disease is characterized by respiratory distress, nervous disorder and reproductive failure^30^, and causes substantial economic losses in the global pig industry^30–32^. PR has been effectively controlled worldwide using a gE-deleted vaccine^33^. However, beginning in 2011, a large-scale outbreak of PR occurred in some Bartha-K61-vaccinated pig herds in China, because PRV has developed new mutations^34,35^. To date, a strategy that completely eradicates PRV is not available, since the virus can establish a latent infection in the peripheral nervous system of the host^36^. Similar to herpes simplex virus type 1 (HSV-1), the genome of PRV is a linear double-stranded DNA composed of approximately 143 kb^30^, and gene expression in PRV is divided into three major groups in a cascade-like manner, as follows: immediately early (IE), early (E) and late (L). EP0, one dominant early gene, is homologous to ICP0 of HSV-1^37^ and ORF61 of varicella-zoster virus (VZV)^38^. The EP0 protein has a RING finger domain at its N-terminus, and this protein executes its regulatory function by interacting with DNA, RNA or other proteins^39^.

Previous studies have identified that a 213 bp segment upstream of the transcription start site (TSS) of EP0 has pan-specific promoter activity. This segment possesses three SP1 binding sites and one IE180 binding site, whereas it lacks the typical TATA element^40,41^. Sp1 is a ubiquitous transcription factor that contains three Cys2His2 zinc finger motifs. It performs its biological function by binding to the consensus site “GGGCGG” in target DNA sequences^42,43^. Notably, Todd and Neidle used *in silico* analyses and revealed a correlation between quadruplex-forming sequences and the Sp1 binding sites in the cis-upstream region of human genes^44^. Recently, a correlation between G4s and SP1 binding sites has also been experimentally confirmed in primate lentiviruses by the Richter group^45^. Given the three Sp1 binding sites in the EP0 promoter, we hypothesized that this G-rich sequence had the potential to fold into a G4.

In this paper, using biochemical and biophysical approaches, we confirmed that the G-rich region within the EP0 promoter forms a parallel-type G4 structure. The resulting G4 structure was bound and stabilized by a small chemical ligand, pyridostatin (PDS). Intriguingly, the Sp1 protein preferentially bound to G4-carrying DNA rather than G4-free DNA. Moreover, based on the results of a luciferase reporter assay, G4 exerted a negative regulatory function towards EP0 promoter activity.

## Results

### G-quadruplex formed within the promoter of EP0

According to a previous study, a 213-bp fragment spanning from −170 bp to +43 bp relative to the TSS of the EP0 gene displays promoter activity *in vitro* and *in vivo*^40,41,46^. We identified a conserved G-rich sequence in this region located from −66 to −26 relative to the TSS, and this G-rich sequence is highly conserved across the genomes of 25 PRV strains (Supplementary Fig. S1). A schematic of this sequence is shown in Fig. 1A. This fragment contains five continuous guanine tracts (red letters) that overlap with three Sp1 binding sites (green oval). By analysing this G-rich sequence with the web-based programme QGRS Mapper^47^, we found that this region displayed a higher potential to form G-quadruplex.

**Figure 2:**
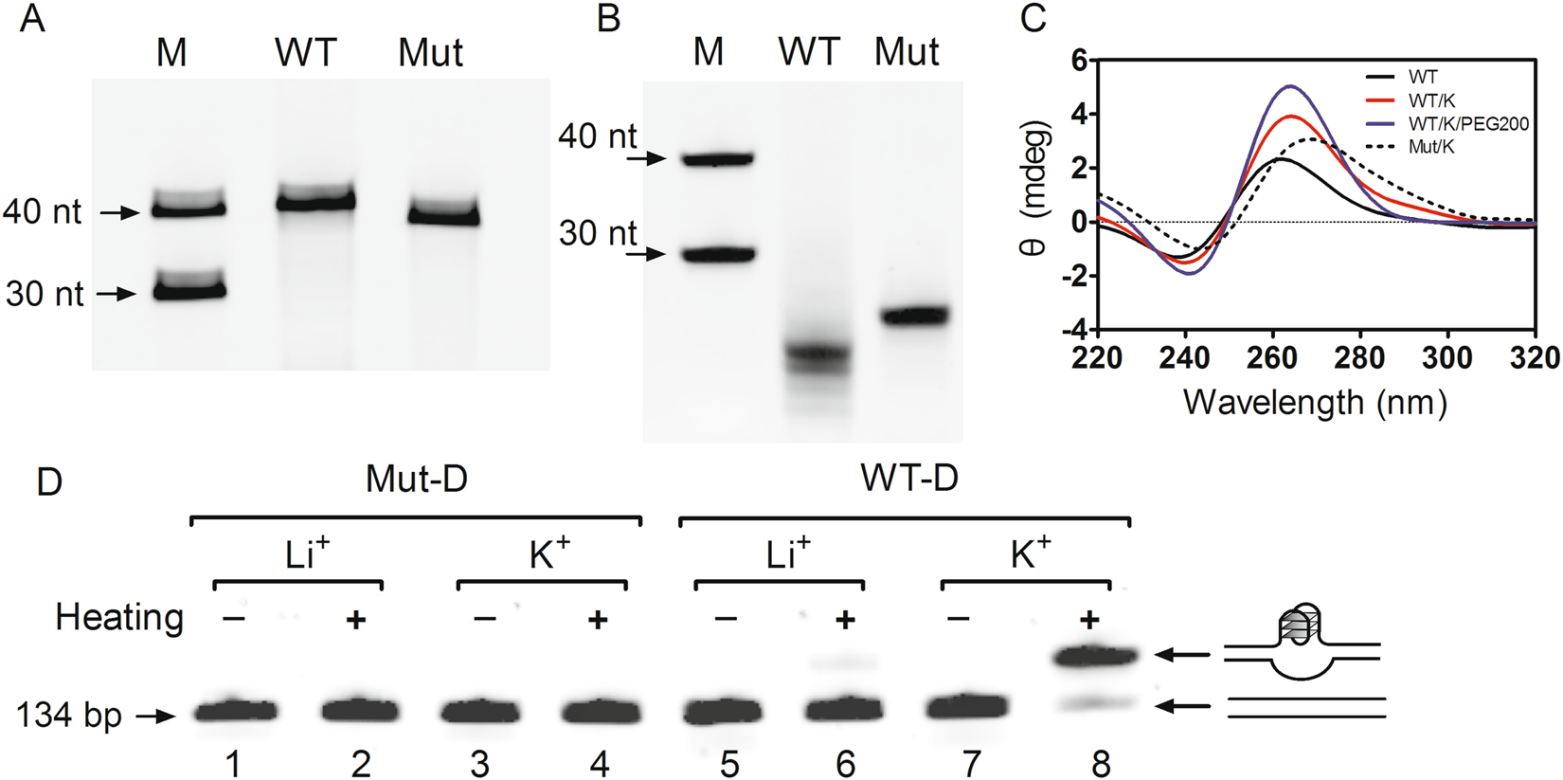
Identification of G4 formation in the G-rich region of the EP0 promoter. (**A**) Single-stranded wild type (WT) and mutated (Mut) DNAs were subjected to denaturing PAGE containing 7 M urea. (**B**) Single-stranded WT and Mut DNAs were subjected to a native PAGE analysis after annealing in 100 mM K^+^ buffer. (**C**) Circular dichroism spectra of WT and Mut ssDNAs. (**D**) Double-stranded wild type (WT-D) and mutated (Mut-D) DNAs were subjected to native PAGE analyses after annealing or were untreated. The drawings on the right indicate linear and G4-carrying DNAs, respectively.

Native polyacrylamide gel electrophoresis (PAGE) and circular dichroism (CD) spectroscopy were initially used to experimentally test whether the G-rich sequence of the EP0 promoter formed a quadruplex. The intramolecular G4 formed by single-stranded DNA exhibits a faster migration rate on native PAGE gels than single-stranded DNA, due to its more compact structure^48–51^. In the present study, synthesized 30-nt and 40-nt poly (T), which do not fold into any secondary structure, were used as markers to assess the relative mobility of the samples. As shown in Fig. 2A, the wild type (WT) ssDNA and its corresponding mutant type (Mut) ssDNA displayed similar migration rates to the 40-nt poly (T) marker on denaturing PAGE gels containing 7 M urea, indicating that these sequences have approximately equal molecular weights. However, when the samples were electrophoresed on native PAGE gels after annealing in K^+^ buffer, the WT ssDNA migrated faster than the 30-nt marker and its counterpart Mut ssDNA (Fig. 2B), indicating that it folded into a more compact intramolecular G4. On the other hand, the Mut ssDNA ran slower than WT ssDNA but faster than 30-nt marker, potentially because the Mut ssDNA formed a hairpin structure after annealing in K^+^ buffer, as predicted by a web-based DNA tool (https://www.idtdna.com/calc/analyzer).

Next, circular dichroism (CD) spectroscopy was performed to confirm the formation of G4s. CD is a popular and valid method used to investigate G4 formation, the topology of the G4 structure, and the effects of small ligands on the thermal stability of G4s^52–55^. As shown in Fig. 2C, the CD spectrum of WT ssDNA was characterized by a negative peak at ~240 nm and a positive peak at ~260 nm, in the presence of the KCl solution, which represented a typical parallel G4 structure^54,56^. Moreover, when PEG200 was added to the K^+^ solution, the peak for WT ssDNA increased compared with the spectrum obtained in the presence of K^+^ alone (red curve) and was much higher than the peak in the spectrum obtained without K^+^ (black curve), consistent with previous reports that the small cosolute PEG200 increases the thermodynamic stability of G4^57–59^.

Because PRV has a double-stranded DNA genome, we next examined whether the double-stranded DNA of the EP0 promoter formed a G4 structure. In this assay, the 5′-FAM-labelled WT dsDNA (WT-D) and the corresponding Mut dsDNA (Mut-D) were subjected to heat denaturation/renaturation and analysed by native PAGE^58,59^. As shown in Fig. 2D, the WT-D containing the putative G4-forming region displayed a slower migrating band above original band when it was subjected to annealing in the presence of a K^+^ solution with PEG200 (Fig. 2D, lane 8). However, the WT-D without annealing displayed only one band, although K^+^ ions were present in the solution (Fig. 2D, lane 7 versus lane 8). In addition, when K^+^ was substituted with Li^+^, only one original band for WT-D was observed (Fig. 2D, lanes 5 and 6), because Li^+^ in principle does not facilitate quadruplex assembly^60^. As a negative control, the Mut-D in which some G bases were mutated to A bases produced only one band (Fig. 2D, lanes 1, 2, 3, and 4). Based on these, the WT dsDNA folded into G4 in the presence of a K^+^ solution.

### Multiple G4s formed in the G-rich region of the EP0 promoter

**Figure 3:**
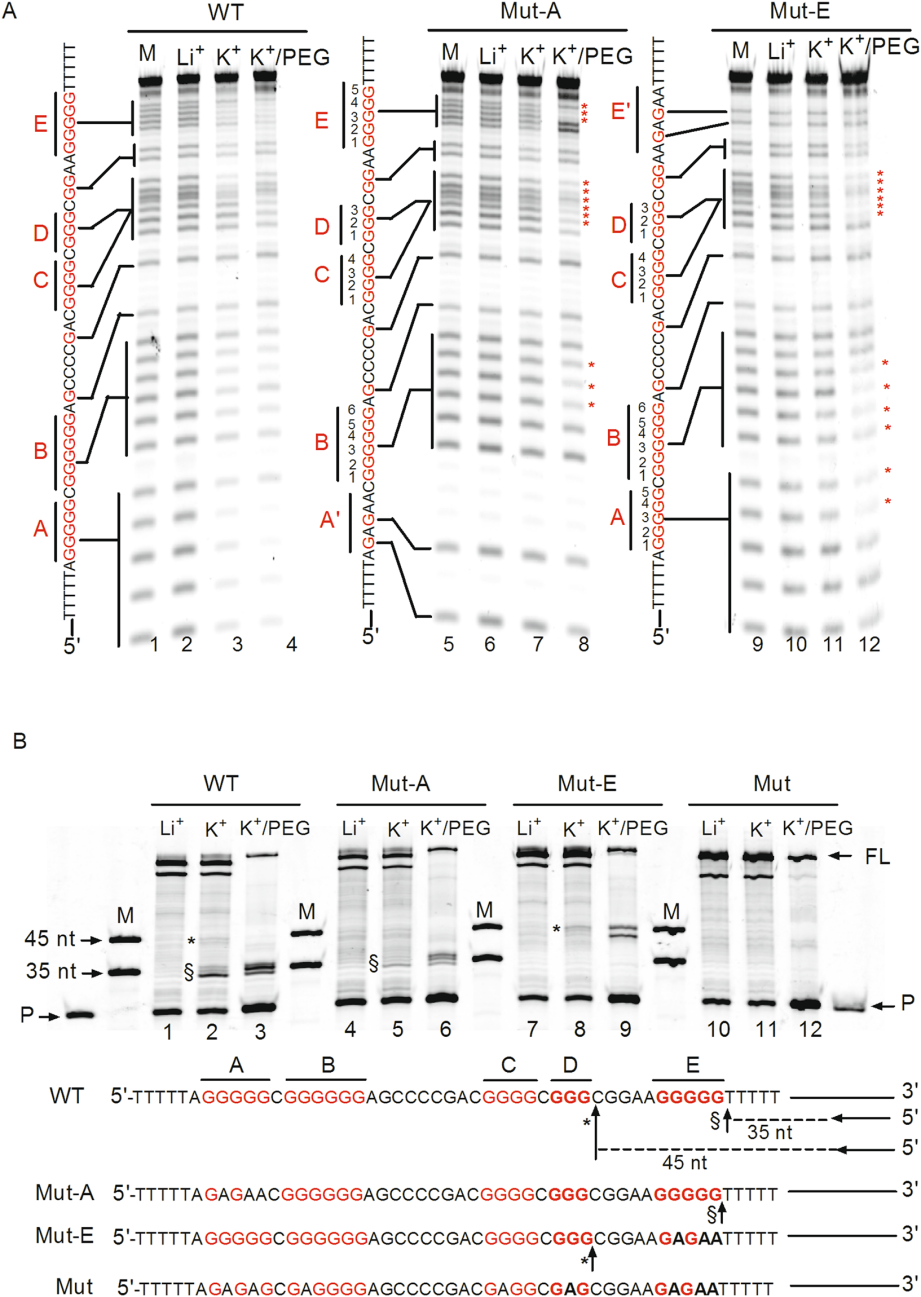
Characterization of quadruplex formation in single-stranded WT, Mut-A and Mut-E DNAs. (**A**) WT DNA, Mut-A with mutations in tract A, and Mut-E with mutations in tract E were subjected to annealing in the presence of LiCl, KCl or KCl plus PEG200, followed by DMS footprinting; the cleavage bands were resolved on denaturing PAGE gels. The base sequences are listed at the left of each gel. Vertical lines indicate the cleavage of G-tracts, the * symbols indicate protected guanines involved in G4 formation. M indicates a marker derived from DMS footprinting without ions. (**B**) Single-stranded template WT, Mut-A, Mut-E and Mut DNAs were annealed with 5′-FAM-labelled primers in the presence of LiCl, KCl or KCl plus PEG200. After the *T*aq polymerase stop assay, the extension products were separated on denaturing PAGE gels containing 7 M urea. P and FL indicate the lanes containing primers and the full-length product, respectively. The stop products were labelled with * and §, respectively. M indicates the synthesized 35-nt and 45-nt oligonucleotides serving as markers. The G-core sequences are listed below the gel and the stop sites are indicated with arrows.

Another concern was which guanines were engaged in quadruplex formation. We aimed to solve this question using a dimethyl sulphate (DMS) footprinting assay. In principle, the N7 of guanine, which is exposed in a DNA duplex or single-stranded DNA, is prone to methylation by DMS and subsequent cleavage by pipedine. Nevertheless, the N7 of guanine would not be attacked by DMS and pipedine when hydrogen bonds formed in the G4 structure. In this experiment, the 5′-FAM-labelled ssDNA shown in Fig. 2A was subjected to heating/annealing in the presence of Li^+^, K^+^ or K^+^/PEG200, followed by the DMS footprinting assay. When Li^+^ ions were applied, G-tracts A, B, C, D, and E displayed distinct cleavage bands (Fig. 3A, lane 2). However, in the presence of K^+^ and K^+^/PEG200, the intensity of the cleavage bands decreased (Fig. 3A, lanes 3 and 4 versus lane 2), implying that the five G-tracts were protected from cleavage and participated in G4 formation.

We introduced G → A mutations in tract A or in tract E of the ssDNA to restrict the polymorphism of G4 formation and further dissect the folding pattern of the five G-tracts in G4. In Li^+^ buffer, the mutated ssDNA species, Mut-A and Mut-E, displayed distinct cleavage patterns (Fig. 3A, lanes 6 and 10). However, in K^+^ buffer, particularly in K^+^/PEG200 buffer, both DNAs exhibited typical protected bands. For Mut-A, the following protected guanines were observed: G3-G4-G5 in tract B; G2-G3-G4 in tract C; G1-G2-G3 in tract D; and G1-G2-G3 in tract E (Fig. 3A, lane 8, labelled with*). For Mut-E, the obviously protected pattern was: G4-G5 in tract A; G1-G2-G3-G4 in tract B; G2-G3-G4 in tract C; and G1-G2-G3 in tract D (Fig. 3A, lane 12, labelled with*). Thus, the G-rich region had the potential to fold into G4, but the G-tracts involved in G4 formation included one G4 produced by tracts A, B, C and D, another G4 composed of tracts B, C, D and E.

Next, we used a *Taq* polymerase stop assay to corroborate the multiple G4 folds. As shown in Fig. 3B, WT DNA showed an apparent 35-nt (symbol §) and a weak 45-nt (symbol *) stop band in K^+^ solution compared to the Li^+^ solution (Fig. 3B, lane 2 versus lane 1), indicating that *Taq* polymerase paused in front of tract E or D, which participated in G4 assembly and hindered the procession of *Taq* polymerase along the template. Furthermore, the 35-nt product obtained in K^+^/PEG buffer was present at a higher level than in K^+^ along (Fig. 3B, lane 3 versus lane 2), which might be attributed to PEG-mediated stabilization of the G4 structure. As expected, Mut-A and Mut-E displayed a 35-nt and 45-nt stop band, respectively (Fig. 3B, lanes 5, 6, 8, and 9), confirming the DMS results shown in Fig. 3A, lanes 8 and 12. As a negative control, the Mut sequence without ability to form a G4 displayed no distinct stop bands (Fig. 3B, lanes 10–12).

### The small ligand PDS promoted and stabilized G4 formation

A ligand targeting G-quadruplexes must fulfil two criteria: (1) the ability to induce or promote G4 formation and (2) the ability to stabilize the formed G4 structure. The small chemical ligand pyridostatin (PDS) has been reported to bind and stabilize the G4s at telomere regions, subsequently inhibiting tumour cell growth^61,62^. Therefore, in this paper, we utilized commercial PDS as an example to investigate the effect of a ligand on the formation and thermostability of G4s in the EP0 promoter. Therefore, fluorescence resonance energy transfer (FRET), the *Taq* polymerase stop assay and native PAGE were conducted.

**Figure 4:**
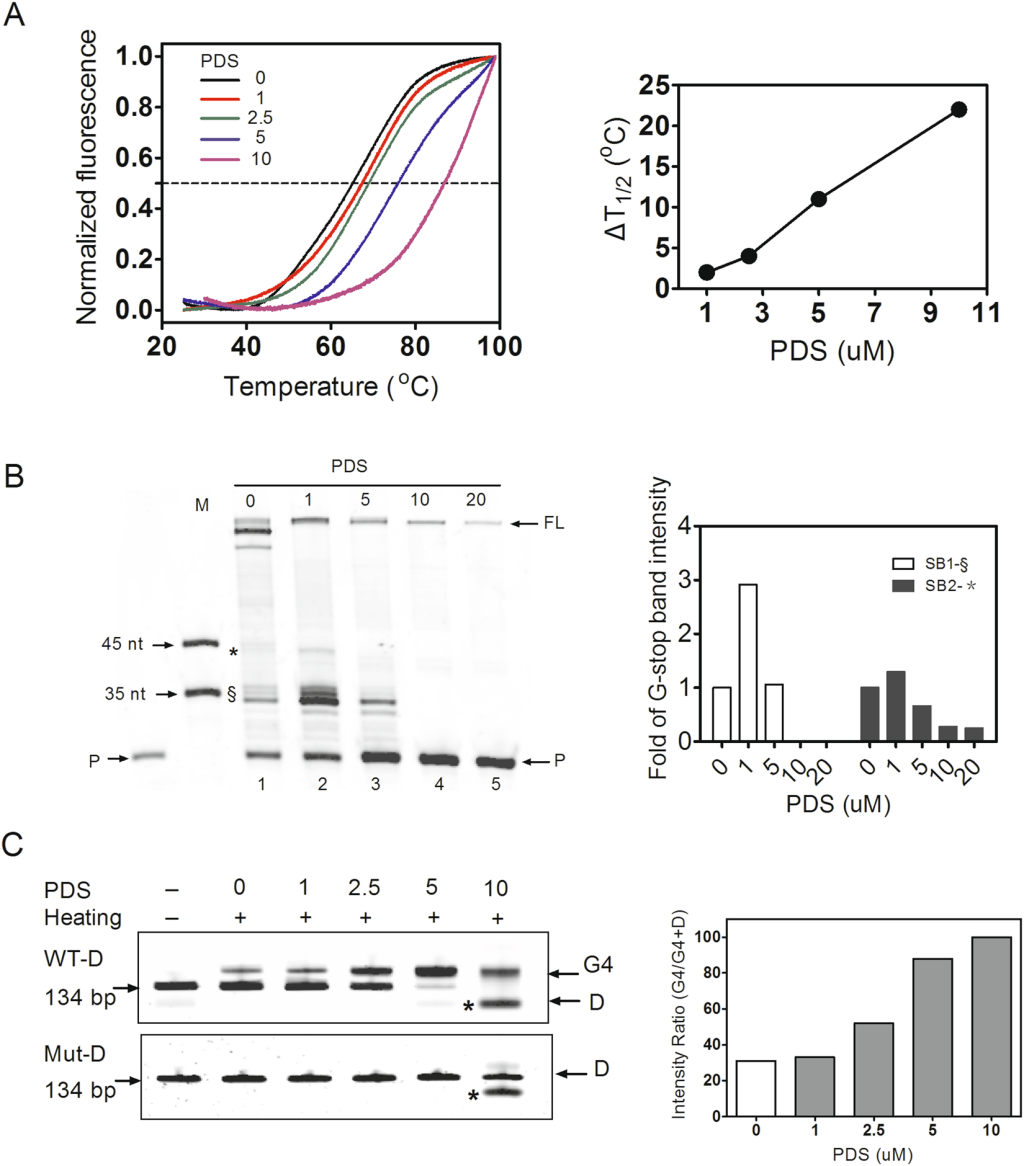
Effect of PDS on the thermal stabilization and folding of G4. (**A**) FRET-melting curves of ssDNAs after annealing and incubation with various amounts of PDS (indicated by the coloured curves). The dashed line indicates the median value between the minimum and maximum fluorescence. (**B**) WT ssDNA was subjected to the *T*aq polymerase assay in the presence of various concentrations of PDS. The quantification of stop bands is shown in the right panel. (**C**) Double-stranded mutated (Mut-D) DNAs were subjected to annealing after the addition of various amounts of PDS, and then the formed G4s were detected by native PAGE analysis. The *symbol denotes an erratic product.

For the FRET assay, the single-stranded DNA was labelled with FAM (as a donor) at the 5′ end and TAMAR (as a quencher) at 3′ end, respectively. Then, the DNA species were annealed to induce G4 formation. When the temperature increased gradually, the donor departed from the quencher, emitting detectable FAM fluorescence (Fig. 4A). The midpoint of the normalized fluorescence intensity between 0 and 1 was referred to as T1/2, which represents the melting temperature. When different amounts of PDS were incubated with the G4-carrying DNA, the T1/2 increased in a PDS concentration-dependent manner. For example, in the presence of 10 μM PDS, the melting temperature increased by 22 °C compared to the sample without PDS (purple curve). Thus, PDS enhanced the thermal stability of G4.

Next, we used the *Taq* polymerase stop assay to assess the effect of PDS on G4 stabilization. WT ssDNAs were annealed in K^+^ buffer to induce G4 formation, followed by extension using *Taq* polymerase. We observed the 35-nt and 45-nt stop products caused by the formation of G4 structures in WT DNA, respectively (Fig. 4B, lane 1). Upon the addition of 1 μM PDS, the amount of the 35-nt stop products increased by approximately 3-fold and the amount of the 45-nt stop product increased by approximately 1.3-fold compared with the sample without PDS (Fig. 4B, lane 2 versus lane 1), indicating that G4 was stabilized by PDS and then hampered the procession of *Taq* polymerase. However, when 5 μM PDS was added to the DNA, the 35-nt stop product decreased to control levels without PDS (Fig. 4B, lane 3) and even disappeared in the presence of greater than 10 μM PDS (Fig. 4B, lane 4 and 5). Meanwhile, the levels of the 45-nt stop products were substantially decreased compared to the control, similar to the full-length products (Fig. 4B, lane 5). A potential explanation for these unexpected results is that the extension activity of *Taq* polymerase was substantially inhibited by excess PDS.

Because the G-rich segment of the EP0 promoter was embedded in double-stranded DNA, we next investigated the effect of PDS on G4 formation under these conditions. In this experiment, various amounts of PDS were added before DNAs were annealed, and then native PAGE was employed to examine G4 formation. As shown in Fig. 4C, the ratio of G4 formation increased gradually upon the addition of increasing concentrations of PDS (Fig. 4C, lanes 1 to 10), as indicated by the appearance of a slower migrating band above the original DNA band (Fig. 4C, lane 1). For mutated dsDNA, only one original band appeared on the gel, regardless of the presence of PDS. When 5 μM PDS was added to the dsDNA, the fraction of G-quadruplex-containing DNA among the total DNA increased to 88% compared with the control without PDS. In particular, in the presence of 10 μM PDS, the fraction reached approximately 100%, indicating that PDS promoted G4 formation during the annealing of dsDNA. However, when 10 μM PDS was added, an erratic band for both WT and Mut DNA was observed (labelled with *). A potential explanation for this finding is that when more PDS bound to the DNA more compact products were generated.

### G4 facilitated Sp1 binding to the EP0 promoter

**Figure 5:**
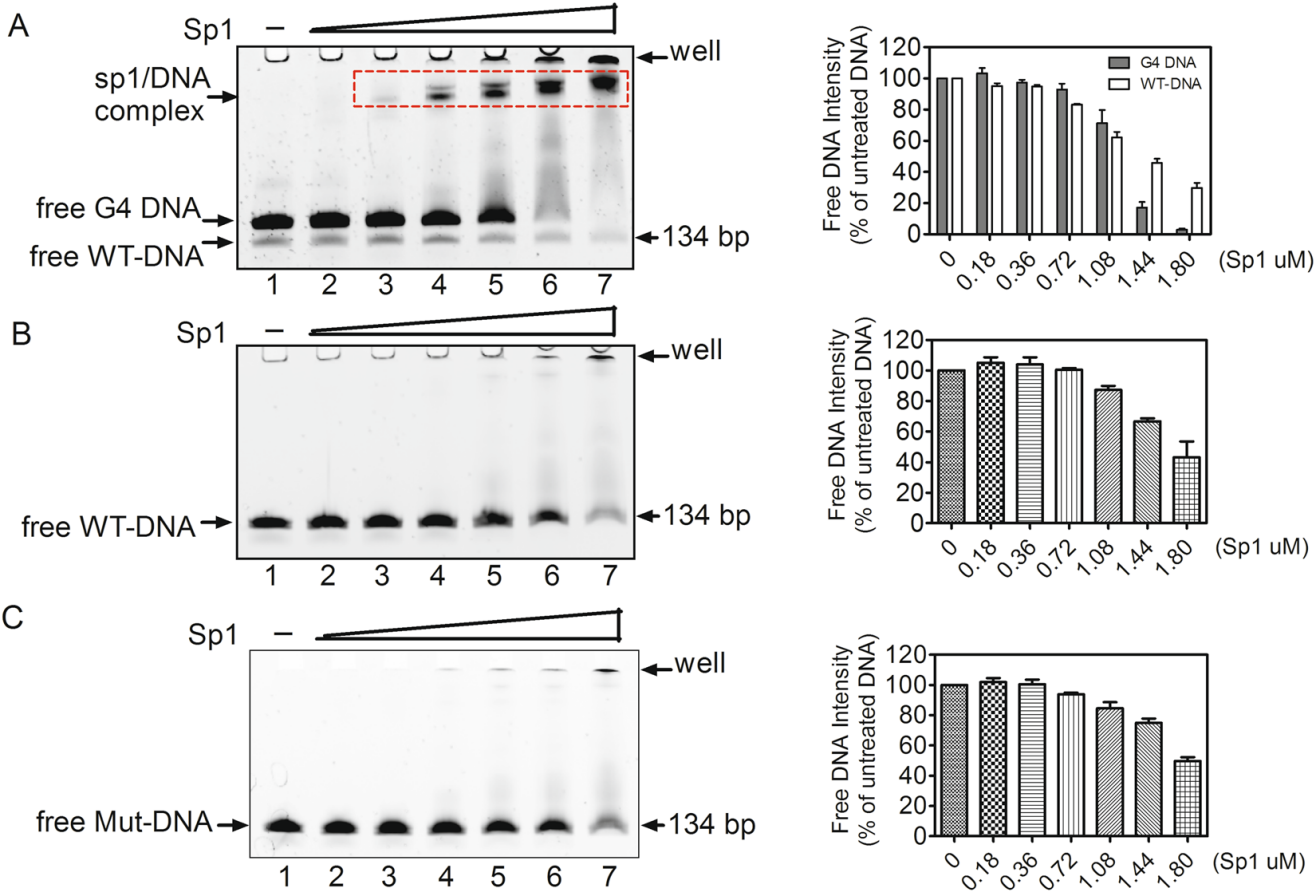
EMSA results showing the binding of MBP-tagged Sp1 to dsDNA with or without G4. (**A**) WT-D subjected to annealing, (**B**) WT-D without annealing, and (**C**) Mut-D subjected to annealing were incubated with the indicated amounts of Sp1, and then the samples were subjected to native PAGE analyses. The dotted box denoted the Sp1 and DNA complex. The quantification of unbound DNA is shown in the right panel.

Because the G4-forming region overlaps with three Sp1 binding sites in the EP0 promoter (Fig. 1A), we intended to explore the correlation between G4 folding and Sp1 binding using EMSAs. As shown in Fig. 5A, 5′-FAMlabeled WT-DNA was subjected to heat denaturation/renaturation to induce G4 formation in a 100 mM K^+^ solution, and then incubated with various amounts of the MBP-tagged, truncated Sp1 protein (a531–770) purified from *E*. *coli*. This fragment consisted of three zinc finger domains, and partial C and D domains^63^. In the absence of Sp1, two DNA bands were observed on the gel (Fig. 5A, lane 1); the upper band represented G4-carrying DNA and the lower band represented the original DNA without G4, as discussed in Fig. 2D. However, in the presence of Sp1, a slower migrating band appeared above the two aforementioned DNA bands (Fig. 5A, lanes 3–7 versus lane 1), implying that Sp1 bound to the DNA and produced a slow-migrating protein-DNA complex. Notably, the formation of this complex was mediated by the Sp1 protein, but not by the MBP tag, because a previous paper has reported that MBP does not show affinity for G4 DNA^64^. In addition, the formation of the complex increased dramatically in a concentration-dependent manner in the presence of increasing concentrations of Sp1 (Fig. 5A, lanes 2–7). Interestingly, as shown in Fig. 5A lanes 6 and 7, the mobility of the G4 DNA was completely shifted by Sp1, while approximately 14% of the free duplex DNA still remained compared to DNA without the addition of Sp1 (Fig. 5A, lane 7 versus lane 1), indicating that Sp1 preferentially bound to G4 DNA compared with duplex DNA. In contrast to annealed WT-DNA that produced G4, the WT-DNA without annealing exhibited weaker affinity for Sp1, as indicated by the weaker band located above the original DNA (Fig. 5B, lane 7). As a negative control, the Mut-DNA containing some G → A mutations to destroy the G4-forming region was not noticeably shifted by Sp1 (Fig. 5C). Furthermore, when the single-stranded DNA with or without G4 was incubated with the Sp1 protein, a similar result was also obtained (Supplementary Fig. S2). Based on these results, we concluded that Sp1 bound to the G4 structure with more affinity than to DNA without G4.

### G4s negatively regulated EP0 promoter activity

**Figure 6:**
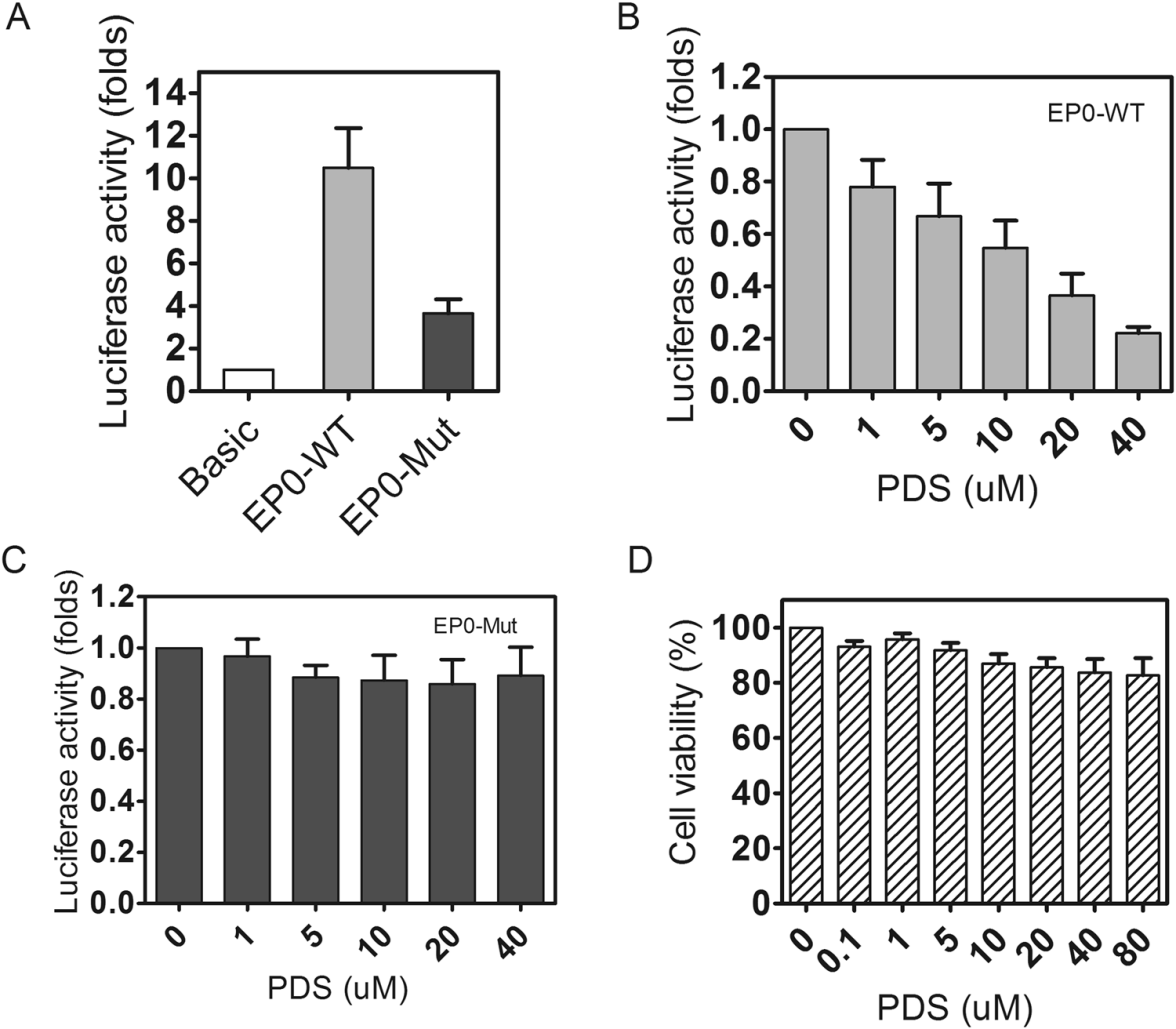
Luciferase reporter assay showing the effects of G4s on the activity of the EP0 and EP0-Mut promoters. (**A**) Luciferase activities of WT and Mut normalized to *Renilla*. (**B**) and (**C**) The effects of PDS on the luciferase activity of EP0-WT and EP0-Mut normalized to total protein content in the cells. (**D**) Results of the MTT assay designed to test the cytotoxicity of PDS towards Vero cells. All values are presented as the means ± s.d., n = 3.

The luciferase reporter assay was performed to investigate the effect of G4s on EP0 promoter activity in a cellular environment. In this experiment, the constructed plasmids, pGL3-EP0-WT or pGL3-EP0-Mut, were cotransfected into Vero cells with pRL-TK. The relative fluorescence intensities of firefly and *Renilla* luciferases were compared to measure the promoter activity. As shown in Fig. 6A, the relative fluorescence intensity of the pGL3-EP0-WT and pGL3-EP0-Mut constructs increased by 10-fold and 3.6-fold, respectively, compared with the pGL-basic construct, suggesting that both EP0-WT and EP0-Mut segments possessed promoter activity. However, the transcriptional activity of EP0-Mut was decreased to approximately 36% of EP0-WT.

Next, we tested the effect of PDS on EP0 promoter activity. In this experiment, the relative luciferase activity was normalized to the total protein content in the cell, rather than the *Renilla* fluorescence intensity, because the *Renilla* fluorescence intensity was obviously affected by PDS (data not shown). In the present study, when increasing amounts of PDS were incubated with transfected Vero cells, the activity of the EP0 promoter decreased gradually in a PDS concentration-dependent manner. For example, when 40 μM PDS was added, the luciferase intensity decreased to 22% of the control without PDS (Fig. 6B). However, as a negative control, the promoter activity of pGL3-EP0-Mut vectors was not substantially affected by PDS (Fig. 6C). Therefore, G4 might act as a negative regulatory element in the EP0 promoter, particularly in the presence of PDS. Notably, the decrease in promoter activity was not caused by cytotoxicity induced by PDS, because PDS nearly did not affect cell viability (Fig. 6D).

## Discussion

In the present study, we identified a conserved G-rich sequence overlapping with three Sp1 binding sites located in the EP0 promoter of PRV. Using biochemical and biophysical methods, we verified that this G-rich sequence formed a G4 structure. In particular, DMS and *Taq* polymerase stop assays indicated that the G-rich region in the EP0 promoter has capacity to form two mutually exclusive G4s. This G4 folding pattern is similar to a previous report for the HIV-1 LTR promoter^26^.

In most cases, G4s perform their regulatory functions by binding to proteins. To date, accumulating evidence has identified some proteins that bind with selective affinity to the G4 DNA, including Pif1 helicase^65^, hnRNP A1^66^, hnRNP A2/B1^67^, Nucleolin^68–70^, Sub1^71^, SLIRP^72^, P53^73^, MAZ^74^, and Sp1^5^. In the present study, the Sp1 protein preferentially bound to G4-carrying DNA compared to DNA without G4 (Fig. 5 and Supplementary Fig. S2). Similar results have been reported by Shankar Balasubramanian and collaborators^5^, who showed that Sp1 binds to the anti-parallel two tetrad quadruplex in the c-KIT promoter. In particular, we speculated that the binding of Sp1 to G4 structures in the EP0 promoter was mainly attributed to its three Cys2His2-type zinc finger motifs. This assumption was verified because the Sp1 protein used in the present study was a truncated protein spanning from amino acids 531 to 770 at its C-terminus, which includes the three intact zinc fingers, but partial C and D domains. A similar scenario has also been reported by Mark Isalan and Sachin D. Patel^75,76^, who showed that the engineered zinc finger protein Gq1 selectively bound to a G-quadruplex formed by single-stranded telomere sequence, but not to the same sequence when in duplex form.

Based on accumulating evidence, G4s that form in promoter regions display regulatory functions, particularly when some small chemical ligands are used to stabilize the formed G4^77–82^. Notably, in the past few years, the number of reports describing G4s in viruses have increased substantially, as reviewed by Richter^83^. In addition, the G4s regulate the viral promoter, particularly with the help of small ligands. For example, the Richter group reported the downregulation of the HIV-1 LTR promoter by the formation of a G4 structure in the promoter^24^. In a DNA virus, hepatitis B virus (HBV), the conserved G-rich motif in the promoter of the preS2/S gene forms a hybrid intramolecular G-quadruplex structure, and the formed G4s positively regulate preS2/S promoter activity with the aid of BRACO^19^ and PDS^24^. Based on the results of the luciferase assay from the present study, G4 negatively regulated the EP0 promoter, particularly in the presence of PDS, which bound to and stabilized the G4 structure that formed in the EP0 promoter (Fig. 4). As a negative control, the transcriptional activity of a G → A mutated promoter that abolished G4 formation decreased to approximately 0.34-fold of the WT promoter (Fig. 6B). The decreased promoter activity of the negative control was inconsistent with some previous findings that the G4-mutated promoter increases the transcriptional activity if G4s in the wild-type promoter act as repressors^78,84^. A potential explanation for this unexpected result was that in the present study, the mutations used to abolish G4 folding also disrupt the Sp1 binding sites in the EP0 promoter, because the G4-forming region overlaps with the three Sp1 binding sites in the EP0 promoter (Fig. 1A).

Sp1 is a critical transcription factor that regulates EP0 promoter activity, since a previous paper has reported that the deletion of Sp1 binding sites in EP0 substantially decreases promoter activity^41^. On the other hand, the results of the luciferase assay in the present study suggested that G4 acts as a repressive modulator to regulate EP0 promoter activity (Fig. 6B). Based on these results, we hypothesized that Sp1 and G4 collaborate to regulate EP0 activity. However, the mechanism underlying the cooperative interaction between Sp1 and G4 requires further investigation.

In conclusion, we reported for the first time that the G-rich region in the EP0 promoter of PRV formed a parallel G4 structure, and the G4 facilitated the binding of Sp1 to the EP0 promoter. Furthermore, with the help of PDS, the G4 exerted negative regulatory effects on promoter activity (Fig. 6B).

## Materials and Methods

The datasets generated during and/or analysed during the current study are available from the corresponding author on reasonable request.

### Preparation of ssDNA and dsDNA

Fluorescein (FAM)-labelled ssDNA and primers were purchased from MBL Beijing. Biotech Co., Ltd. (Beijing, China); other ssDNA and primers were purchased from Sangon Biotech (Shanghai, China). The wild type FAM-labelled dsDNA containing the core G-rich sequence (AGGGGGCGGGGGGAGCCCCGACGGGGCGGGCGGAAGGGGG) was obtained by polymerase chain reaction (PCR) using the Bartha-K61 genome (a gift from Jiang Wang, The College of Animal Sciences and Veterinary Medicine, Henan Agricultural University) as the template, and oligonucleotides (5′-FAM-AGGGCCCGCTTCCCACGA and 5′-GGTGCCCGGCCCCTTTGTT) were used as primers. A synthetic dsDNA fragment of the EP0 promoter containing the mutated G-rich sequence (AGAGAGCGAGGGGAGCCCCGACGAGGCGAGCGGAAGAGAG) was inserted into the TA cloning site of pMD-19-T simple plasmid (TaKaRa Biotech, Dalian, China) to prepare the mutated FAM-labelled dsDNA. This construct was used as a template for PCR, and the primers were the same as used to prepare wild type PCR product.

### Analysis of sequence conservation

The EP0 promoter sequences located from −74 to −23 were derived from the National Center for Biotechnology Information website (www.ncbi.nlm.nih.gov). Multiple sequence alignment was conducted with MEGA6 software^85^.

### Native polyacrylamide gel electrophoresis (PAGE)

Commercially synthesized wild type and mutated type ssDNAs (Table S1) were diluted to 1 μM in 50 mM lithium cacodylate buffer (pH 7.4) containing 40% (w/v) PEG200 and 100 mM KCl. Wild type and mutated type 5′-FAM labelled dsDNAs obtained by PCR were diluted to the final concentration of 1 μM in 50 mM Tris·HCl (pH 7.4) buffer containing 40% (w/v) PEG 200 and 100 mM KCl or 100 mM LiCl. Both ssDNA and dsDNA samples were heated to 95 °C for 5 min, and then slowly cooled to room temperature at a rate of 0.01 °C/s. Afterwards, the samples were loaded on 8% native polyacrylamide gels containing 100 mM KCl and 40% (w/v) PEG 200, and electrophoresed at 4 °C and a rate of 8 V/cm; the gel was then scanned using an Amersham Imager 600 (GE Healthcare).

### Circular dichroism (CD) spectroscopy

Wild type and mutated type ssDNAs (Table S1) were diluted to the final concentration of 4 μM in 50 mM lithium cacodylate buffer (pH 7.4) containing 100 mM KCl, unless specified otherwise. The samples were heated at 95 °C for 5 min and then slowly cooled to room temperature at a rate of 0.01 °C/s. Afterwards, CD spectra were recorded on a Chirascan-plus Circular Dichroism Spectrophotometer (Applied Photophysics) from 220 to 320 nm at 25 °C with 0.5-mm path length and 1-nm bandwidth. The spectra for all samples were baseline-corrected with buffer and represented the average of three runs.

### Dimethyl sulphate (DMS) footprinting

DMS footprinting was conducted using previously described methods^59^, with some modifications. Briefly, 5′-FAM labelled ssDNAs (Table S1) were diluted to concentration of 0.2 μM in 50 mM Tris·HCl (pH 7.4) buffer containing 100 mM LiCl or 100 mM KCl plus 40% (w/v) PEG 200. After heating at 95 °C for 5 min and slowly cooling to 25 °C at 0.01 °C/s, 150 μL of water were added to the annealed DNA sample to obtain a total volume of 200 μL. Then, the samples were incubated with 4 μL of 10% (v/v) dimethyl sulphate (DMS) in ethanol at room temperature for 6 min. The reaction was stopped by the addition of 200 μL of stop buffer (0.1 M β-mercaptoethanol and 20 μg of sperm DNA). After phenol/chloroform extraction and ethanol precipitation, the oligonucleotides were dissolved in 50 μL of water and mixed with 50 μL of 20% (v/v) piperidine in water. The samples were heated at 90 °C for 30 min and dried in a Concentrator Plus (Eppendorf, Germany); the pellets were resuspended in 100 μL water and the sample was dried again. The precipitated DNAs were dissolved in 95% (v/v) deionized formamide in water containing 5 mM EDTA, heat-denatured at 95 °C for 5 min and resolved on a denaturing 16% polyacrylamide gels containing 7 M urea. Gels were scanned using an Amersham Imager 600 (GE Healthcare).

### *Taq* Polymerase stop assay

The *Taq* polymerase stop assay was conducted using previously described methods^45^, with some modifications. Briefly, 1 μM template and 1.2 μM primer (Table S1) were heated at 95 °C for 5 min in buffer containing 50 mM LiCl, 50 mM KCl or 50 mM KCl plus 40% (w/v) PEG200, and then cooled to 25 °C at a rate of 0.01 °C/s. The indicated concentrations of PDS were added. Primer extension was performed with 2.5 U of *Taq* DNA polymerase (Thermo Scientific Fermentas) at 60 °C for 30 min. Reactions were stopped by ethanol precipitation, and the extension products were resolved on a denaturing 16% polyacrylamide gel containing 7 M urea. The gel was scanned with an Amersham Imager 600 (GE Healthcare).

### Fluorescence resonance energy transfer (FRET) melting assay

Oligonucleotides (Table S1) were diluted to a final concentration of 0.4 μM in 50 mM lithium cacodylate buffer (pH 7.4) containing 50 mM KCl. After heating at 95 °C for 5 min and slowly cooling to 25 °C at a rate of 0.01 °C/s, the indicated concentrations of PDS (Sigma) were added to the sample and incubated at 25 °C for 30 min. After equilibration at 25 °C for 5 min, FRET melting curves were constructed as previously described^86^ by monitoring FAM fluorescence on a QuantStudio^TM^ 7 Flex Real-Time PCR System (Life Technologies) as the temperature increased to 99 °C at a rate of 0.015 °C/s.

### Electrophoretic mobility shift assay (EMSA)

The 5′-FAM-labelled dsDNA or ssDNA (Table S1) was diluted to 0.5 μM in 50 mM Tris·HCl (pH 7.4) buffer containing 100 mM KCl and 40% (w/v) PEG 200. After heating at 95 °C for 5 min and slowly cooling to 25 °C at a rate of 0.01 °C/s, various amounts of purified MBP-tagged Sp1 were incubated with the annealed DNA in binding buffer (12.5 mM Hepes-KOH, pH 7.5, 6.25 mM MgCl_2,_ 10% (v/v) glycerol, 0.05% (v/v) NP-40, 5 µM ZnSO_4_, 50 mM KCl, and 50 µg/ml BSA). The binding reaction was performed at 4 °C for 1 h, then the samples were loaded on an 8% native polyacrylamide gel containing 100 mM KCl and 40% (w/v) PEG 200, electrophoresed at 4 °C at a rate of 8 V/cm, and the gels were imaged using an Amersham Imager 600 (GE Healthcare).

### Plasmid construction

The EP0 promoter fragments (ranging from −170 to +43 relative to TSS) were obtained by PCR using the Bartha-K61 genome as template, and the oligonucleotides 5′-CACGGTACCAGAGCGGGGGATCCGCA and 5′-ACCAAGCTTGGTGTCGAGGGCCCCGTT were used as the primer pair to construct pGL3-EP0. The PCR products were digested with HindIII and KpnI (NEB, USA) and purified using the Wizard SV Gel and PCR clean-Up System (Promega, USA). The purified DNA fragments were then inserted into the HindIII and KpnI sites of pGL3 (Promega, USA). A synthetic DNA fragment of the EP0 promoter in which the core G-rich sequence was replaced with AGAGAGCGAGGGGAGCCCCGACGAGGCGAGCGGAAGAGAG was used as the template and PCR was performed as described for pGL3-EP0 to construct the pGL3-EP0-Mut plasmid. This mutated fragment was also inserted into the HindIII and KpnI sites of pGL3. All constructs were sequenced.

### Transfection and luciferase assay

Vero cells were cultured on 24-well plates at a density of 1 × 10^5^ cells/well in Dulbecco’s Modified Eagle’s Medium (DMEM) supplemented with 10% foetal bovine serum at 37 °C, in a humidified atmosphere containing 5% CO_2_. When the cells reached approximately 80% confluence, cells in each well were cotransfected with 760 ng of the pGL3-EP0 or pGL3-EP0-Mut plasmid and 40 ng of the pRL-TK reference plasmid using the FuGene HD Transfection Reagent (Promega, USA); the pGL3-Basic vector was used as a negative control. After growing for 150 min, the cells were washed twice with PBS, and then the media were replaced with DMEM containing the indicated amounts of PDS in the dark. After culture for 24 h, the luciferase activity was determined using the Dual Luciferase Assay kit (Promega, USA), according to the manufacturer’s instructions. The ratios of firefly/*Renilla* luciferase activities were calculated and normalized to the levels observed in cells transfected with the basic vectors, which were set to 1.0.

Cells were lysed in RIPA buffer (50 mM Tris-HCl pH 7.2, 150 mM NaCl, 1% Igepal, and 0.1% SDS) and the protein concentration was determined using the BCA assay (Thermo Scientific Pierce, USA) to measure the luciferase activity in cells cultured with PDS. The luciferase activity was normalized to the total protein content. All experiments were performed in biological triplicates.

### MTT assay

The MTT (3-(4,5-dimethylthiazol 2-yl)−2,5-diphenyltetrazolium bromide) assay was conducted as previously described, with some modifications. Briefly, Vero cells were cultured on 96-well plates at a density of 1 × 10^4^ cells/well in Dulbecco’s Modified Eagle’s Medium (DMEM) supplemented with 10% foetal bovine serum at 37 °C in a humidified atmosphere containing 5% CO_2_. When the cells reached approximately 60% confluence, the media was replaced with 200 μL of fresh DMEM containing different concentrations of PDS. After a 20 h incubation, 20 μL of a freshly dissolved solution of MTT (5 mg/mL in PBS) were added to each well. After a 4 h incubation, 150 μl of DMSO were added to each well to dissolve the MTT crystals. Finally, the absorbance was recorded at 490 nm with a Multi-Mode Microplate Reader (Thermo Scientific, USA). All experiments were performed in biological triplicates.

## Electronic supplementary material

Dataset 1
