# Supplementary material for: Identification and characterization of G-quadruplex formation within the EP0 promoter of pseudorabies virus
Source: Sci Rep. 2018 Sep 19;8:14029. doi: 10.1038/s41598-018-32222-7 (PMC6145870; doi:10.1038/s41598-018-32222-7)
Supplement: Supplementary file 1 — Dataset 1 [file 41598_2018_32222_MOESM1_ESM.pdf]

Identification and characterization of G-quadruplex formation within the EP0 promoter of pseudorabies virus

Jiang-Nan kong[1], Chao Zhang[1*], Yan-Ce Zhu[1], Kai Zhong[1], Jiang Wang[1], Bei-Bei Chu[1] & Guo-Yu Yang[1*]

[1] College of Animal Science and Veterinary Medicine, Henan Agricultural University, Zhengzhou 450002, Henan Province, PR China.

*To whom correspondence should be addressed:
College of Animal Science and Veterinary Medicine, Henan Agricultural University, Zhengzhou 450002, Henan Province, PR China.
Tel.: +86 (371) 56990163; fax: +86 (371) 55369208.
E-mail: C.Z. (chaozhang@henau.edu.cn, lbandeng@126.com) or G.Y. (haubiochem@163.com)

GenBank Accession number	Aligned sequence
KU900059.1	ACGGCCCGAGGGGGCGGGGGGAGCCCCGACGGG-GCGGGCGGAAGGGGGCGT
KJ717942.1	ACGGCCCGAGGGGGCGGGGGGAGCCCCGACGGG-GCGGGCGGAAGGGGGCGT
JQ809330.1	ACGGCCCGAGGGGGCGGGGGGAGCCCCGACGGG-GCGGGCGGAAGGGGGCGT
JQ809329.1	ACGGCCCGAGGGGGCGGGGGGAGCCCCGACGGG-GCGGGCGGAAGGGGGCGT
JQ809328.1	ACGGCCCGAGGGGGCGGGGGGAGCCCCGACGGG-GCGGGCGGAAGGGGGCGT
JF797218.1	ACGGCCCGAGGGGGCGGGGGGAGCCCCGACGGG-GCGGGCGGAAGGGGGCGT
JF797217.1	ACGGCCCGAGGGGGCGGGGGGAGCCCCGACGGG-GCGGGCGGAAGGGGGCGT
KU198433.1	ACGGCCCGAGGGGGCCGGGGGAGCCCCGACGGG-GCGGGCGGAAGGGGGCGT
KT983811.1	ACGGCCCGAGGGGGCGGGGGGAGCCCCGACGGG-GCGGGCGGAAGGGGGCGT
KT983810.1	ACGGCCCGAGGGGGCGGGGGGAGCCCCGACGGG-GCGGGCGGAAGGGGGCGT
JF797219.1	ACGGCCCGAGGGGGCGGGGGGAGCCCCGACGGG-GCGGGCGGAAGGGGGCGT
KM189913.1	ACGGCCCGAGGGGGCGTGGGGAGCCCCGACGGG-GCGGGCGGAAGGGGGCGT
KT809429.1	ACGGCCCGAGGGGGCGTGGGGAGCCCCGACGGG-GCGGGCGGAAGGGGGCGT
KU360259.1	ACGGCCCGAGGGGGCGTGGGGAGCCCCGACGGG-GCGGGCGGAAGGGGGCGT
KM189912.1	ACGGCCCGAGGGGGCGTGGGGAGCCCCGACGGG-GCGGGCGGAAGGGGGCGT
KT824771.1	ACGGCCCGAGGGGGCGTGGGGAGCCCCGACGGG-GCGGGCGGAAGGGGGCGT
KM189914.3	ACGGCCCGAGGGGGCGTGGGGAGCCCCGACGGG-GCGGGCGGAAGGGGGCGT
KM061380.1	ACGGCCCGAGGGGGCGTGGGGAGCCCCGACGGG-GCGGGCGGAAGGGGGCGT
KJ789182.1	ACGGCCCGAGGGGGCGTGGGGAGCCCCGACGGG-GCGGGCGGAAGGGGGCGT
KC981239.1	ACGGCCCGAGGGGGCGTGGGGAGCCCCGACGGG-GCGGGCGGAAGGGGGCGT
KU057086.1	ACGGCCCGAGGGGGCGTGGGGAGCCCCGACGGG-GCGGGCGGAAGGGGGCGT
KP722022.1	ACGGCCCGAGGGGGCGTGGGGAGCCCCCACGGG-GCGGGCGGAAGGGGGCGT
KP257591.1	ACGGCCCGAGGGGGCGTGGGGAGCCCCGACGGG-GCGGGCGGAAGGGGGCGT
KP098534.1	ACGGCCCGAGGGGGCGTGGGGAGCCCCGACGGGCGCGGGCGGAAGGGGGCGT
KU315430.1	ACGGCCCGAGGGGGCGTGGGGAGCCCCGACGGG-GCGGGCGGAAGGGGGCGT

Supplementary Figure S1. Premade sequence alignment of the core G-rich sequences in EP0 promoter across 25 PRV strains. The EP0 promoter sequences located from -74 to -23 were derived from the National Center for Biotechnology Information Web site (www.ncbi.nlm.nih.gov), and then aligned by MEGA6 software, the conserved G-rich region were shown in red.

Table S1 Oligonucleotides sequence used in this paper for PAGE, EMSA, CD, DMS, Taq polymerase and FRET

Technique	Oligo Name	Oligo Sequence (5'-3')
PAGE EMSA	WT	[FAM]-AGGGGGCGGGGGGAGCCCCGACGGGGCG GGCGGAAGGGGG
	Mut	[FAM]-AGAGAGCGAGGGGAGCCCCGACGAGGCG AGCGGAAGAGAG
CD	WT	AGGGGGCGGGGGGAGCCCCGACGGGGCGGGCGG AAGGGGG
	Mut	AGAGAGCGAGGGGAGCCCCGACGAGGCGAGCGG AAGAGAG
DMS	WT	[FAM]-TTTTTAGGGGGCGGGGGGAGCCCCGACGG GGCGGGCGGAAGGGGGTTTTT
	Mut-A	[FAM]-TTTTTAGAGAACGGGGGGAGCCCCGACGG GGCGGGCGGAAGGGGGTTTTT
	Mut-E	[FAM]-TTTTTAGGGGGCGGGGGGAGCCCCGACGG GGCGGGCGGAAGAGAATTTTT
Taq polymerase stop assay	WT	TTTTTAGGGGGCGGGGGGAGCCCCGACGGGGCG GGCGGAAGGGGGTTTTTCGCACTGAGCGAAGATA CGGAGCCACGCCA
	Mut-A	TTTTTAGAGAACGGGGGGAGCCCCGACGGGGCG GGCGGAAGGGGGTTTTTCGCACTGAGCGAAGATA CGGAGCCACGCCA
	Mut-E	TTTTTAGGGGGCGGGGGGAGCCCCGACGGGGCG GGCGGAAGAGAATTTTTCGCACTGAGCGAAGATA CGGAGCCACGCCA
	Mut	TTTTTAGAGAGCGAGGGGAGCCCCGACGAGGCG AGCGGAAGAGAGTTTTTCGCACTGAGCGAAGATA CGGAGCCACGCCA
	Primer	[FAM]-TGGCGTGGCTCCGTATCTTCGCTCAG
FRET	WT	[FAM]-AGGGGGCGGGGGGAGCCCCGACGGGGCG GGCGGAAGGGGG-[TAMRA]

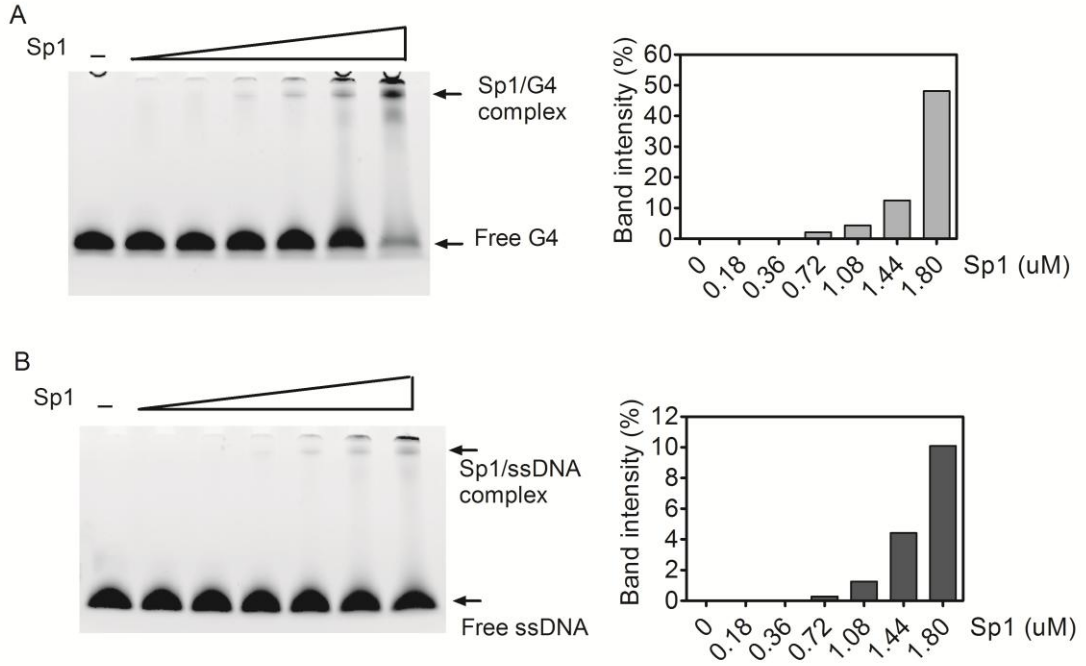

Supplementary Figure S2. EMSA assay to determine the binding of MBP-tagged Sp1 to the single-stranded DNA with or without G4. Various amounts of Sp1 were incubated with 5'-FAM labeled ssDNA subjected to anneal (A) or not (B), then the samples were analyzed through native PAGE. The quantification of SP1/G4 or Sp1/ssDNA complex band intensity was on the right panel.
